# Polyvinyl Alcohol/Calcium Carbonate Nanocomposites as Efficient and Cost-Effective Cationic Dye Adsorbents

**DOI:** 10.3390/polym12102179

**Published:** 2020-09-24

**Authors:** Davoud Jahani, Amin Nazari, Jaber Ghourbanpour, Amir Ameli

**Affiliations:** 1 Faculty of Engineering, University of Bonab, Bonab 55517, Iran; davoud@ubonab.ac.ir; 2 Faculty of Engineering, University of Maragheh, Maragheh 55181, Iran; amin1371n@gmail.com (A.N.); jaber_gh151@yahoo.com (J.G.); 3 Plastics Engineering Department, University of Massachusetts Lowell, Lowell, MA 01854, USA

**Keywords:** dye removal, nanocomposite, calcium carbonate, adsorption, kinetics, isotherm

## Abstract

A novel polyvinyl alcohol (PVA)/calcium carbonate-based double-layer cationic dye adsorbent was developed. Polyvinyl alcohol (50 wt %) and calcium carbonate (50 wt %) were used together with borax as a cross-linking agent. The nanocomposite was prepared using only water, without the need for any toxic solvent or hazardous chemical. The final samples were obtained by the solvent casting method. The nanocomposite adsorbent was characterized using a Fourier transform infrared (FTIR) spectroscope and a scanning electron microscope (SEM). The adsorption performance on two cationic dyes, i.e., methylene blue and safranin was studied. Dye adsorption was quantified by measuring the nanocomposite swelling, contact time, and dye concentration. Pseudo first-order and pseudo second-order kinetic models as well as intraparticle diffusion model were used to model the adsorption kinetics. Moreover, the isotherm dye adsorption was investigated by Langmuir and Freundlich models. The results revealed that the developed nanocomposite has relatively high adsorption efficiency and short adsorption time and retains its performance after several successive absorption–desorption processes. The results also showed that the pseudo-second-order model best describes the adsorption kinetics, and the Freundlich isotherm model has a better compatibility with the experimental data. Finally, an adsorption mechanism was proposed for the dye removal process. The developed PVA/CaCO_3_ nanocomposite can be potentially used for efficient dye removal in wastewater treatments.

## 1. Introduction

Industrial advances are usually associated with industrial effluents, which disrupt the environment, especially aquatic environments [1,2]. Dye pollution is a complex problem due to the dyes’ molecular structure and their resistance to biodegradation [3,4]. Cationic dyes, which are made of organic compounds or hydrochlorides with the general formula of OH–R–NH_2_, are widely used in the textile industry due to their high dyeing values. They are also used for dyeing silk, paper, leather, and production of inks [5,6].

Recently, the use of nanomaterials such as titanium dioxide [7], silicon dioxide silver [8], and zinc oxide [9] as paint absorbers has received particular attention. Nanomaterials have a large surface area at a given volume, which allows them to absorb large amounts of paint in a short time. However, there have been significant concerns about heavy metal nanoparticles entry into water due to the use of some nanomaterials [10,11]. Recently, polyvinyl alcohol (PVA)-based hydrogel materials have also been used as efficient dye absorbers [12,13,14,15,16]. Mok et al. prepared PVA hydrogel as an agent to remove dye from wastewater using methylene blue (MB) in aqueous solution [16]. Glutaraldehyde (GA) was used as a crosslinking agent and the hydrogel was characterized in terms of morphology, swelling, and adsorption. It was found that GA content and solution pH have a considerable effect on the adsorption capacity of PVA hydrogel [16]. In a separate study [17], Papancea et al. examined PVA-scleroglucan, PVA-zein, and PVA-cellulose in combination with different types of dye such as MB, crystal violet (CV), and Congo red (CR) [17]. It was revealed that PVA-scleroglucan hydrogel has the best dye-adsorption efficiency.

Very recently, Ghorbanpour et al. [18] reported a nanocomposite hydrogel adsorbent composed of PVA and chitin nanofibers. They used PVA and Iron (III) chloride in the presence of chitin nanofibers as the matrix and cross-linking agent, respectively. Their study revealed that the PVA/chitin nanocomposite hydrogel has a high potential for anionic dye absorption, and the uptake capacity can reach 92% at pH = 4, in the presence of chitin nanofibers. Li et al. [19] also used graphene oxide (GO) in a PVA-based hydrogel to improve hydrogel uptake capacity. To evaluate its effectiveness, the crystal structure, synthetic swelling, and MB absorption of PVA/GO hydrogels were systematically investigated. Their study showed that the interaction of PVA and MB includes both chemical and physical absorption, and GO can lead to significant improvement in MB absorption [19].

The reported nanomaterials in the fabrication of nanocomposite hydrogels are usually expensive or difficult to scale up. As nanocomposites have shown great promise in enhancing the dye absorption of hydrogels, there is a need to find affordable, scalable, and harmless nanomaterials. Calcium carbonate (CaCO_3_) is an inexpensive and widely available mineral that is easily found in rocks all over the world. It is also found in eggshells and animal membranes such as snails and crabs and can be produced artificially too. Calcium carbonate is used as a filler in various industries [20,21]. CaCO_3_ as a low-cost adsorbent [22] is commonly used in heavy metal removal [23], oil spills [24], and dye removal from water [25]. CaCO_3_ particles have a high surface area [26,27] and can be coated for improved performance [27,28].

In this work, we systematically investigate the dye removal capability of PVA/CaCO_3_ nanocomposites. PVA/CaCO_3_ nanocomposites were fabricated by solution casting and an air drying process. A solution of PVA and CaCO_3_ in water was prepared by ultrasonication in the presence of sodium borate (borax) as the PVA’s crosslinking agent. The gels were then cast and air dried to obtain the test samples. Scanning electron microscopy (SEM) and Fourier transform infrared (FTIR) spectroscopy were used to characterize the nanocomposite’s microstructure and chemical bonding. Dye removal efficiency was studied by measuring the nanocomposite swelling and dye removal percentage as a function of time, dye concentration, and solution pH. Using pseudo first-order and pseudo second-order kinetic models and intraparticle diffusion model, the adsorption kinetics were investigated. The isotherm dye adsorption was also studied by the Langmuir and Freundlich models. Finally, an adsorption mechanism is proposed for the dye-removal process of the developed nanocomposite.

## 2. Experimental

### 2.1. Materials

PVA with a degree of hydrolysis greater than 89% and a molecular weight of 72,000 g/mol was obtained from Sigma Aldrich (Merch KGaA, Darmstadt, Germany). Calcium carbonate was purchased from Shell, Indore, India. Methylene blue (MB) dye with a purity of 95% and a molecular weight of 319.86 g/mol were purchased from Merck KGaA, Darmstadt, Germany. Safranin (SF) with a dye content of 85% and a molecular weight of 350.84 g/mol was purchased from Sigma-Aldrich (Merch KGaA, Darmstadt, Germany). All the materials were used as received.

### 2.2. Fabrication of Nanocomposites

**Figure 1:**
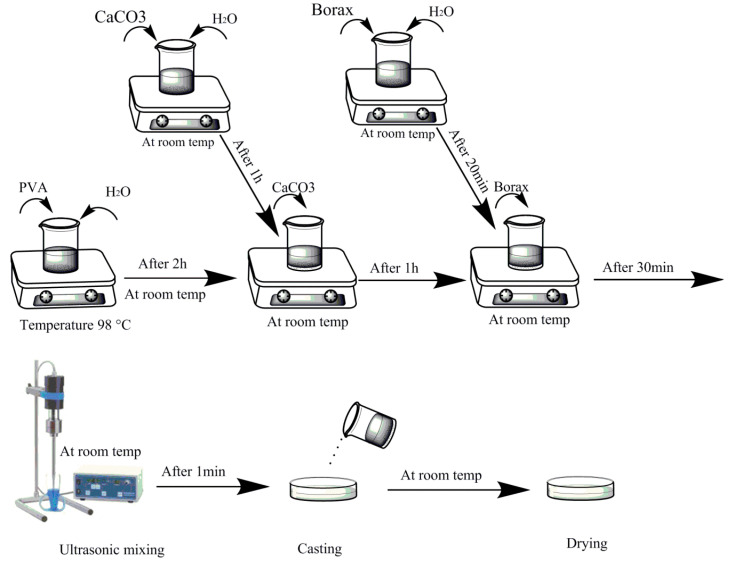
Schematic diagram of experimental procedures to prepare polyvinyl alcohol (PVA)/CaCO_3_ nanocomposites.

Figure 1 shows a schematic diagram of experimental procedure to fabricate the nanocomposite hydrogels. In the preparation of PVA/CaCO_3_ nanocomposites, at first 0.5 g of PVA was mixed with 20 mL of distilled water at 98 °C, and they were kept on the mixer for two hours to obtain the PVA/water solution. Also, separately, 0.5 g of calcium carbonate was mixed with 20 mL distilled water for one hour at room temperature to obtain a homogenous suspension of calcium carbonate in the water. Prepared calcium carbonate suspension was gently added to the PVA solution and mixed for one hour. Then, 0.05 g of borax, as a cross-linking agent, was dissolved in 10 mL distilled water, and then gently added to the prepared nanocomposite solution, and an ultrasonic mixer was used to homogenize the solution. Finally, the resultant solution was cast in a suitable container and left at room temperature for 72 h to dry.

### 2.3. Characterization

#### 2.3.1. Fourier Transform Infrared (FTIR) Spectroscopy

FTIR spectroscopy was used to study the chemical groups of the materials. FTIR spectra of PVA/CaCO_3_ nanocomposites, PVA, and calcium carbonate particles were recorded using an FTIR spectrometer (IR affinily-1s, Shimadzu, Tokyo, Japan) in the frequency range of 4000–500 cm^−1^.

#### 2.3.2. Scanning Electron Microscopy (SEM)

Field-emission scanning electron microscopy (FESEM) (MIRA3 FEG-SEM, Tescan, Kohoutovice, Czech) was used to study the dispersion state and distribution of nanoparticles in the matrix.

#### 2.3.3. Dye Removal and Adsorption Efficiency

Dye removal percentage as a fraction of initial dye concentration can be defined as:

(1) $$Dye\;Removal\;\left(\%\right)=\;\left(\frac{C_{0}-C_{t}}{C_{0}}\right)\times 100\%$$

where *C*_0_ (mg/L) and *C_t_* (mg/L) are the initial dye concentration and dye concentration at time *t*, respectively [29]. The adsorption efficiency or adsorption capacity at equilibrium can be defined when the dye concentration of the solution reaches equilibrium. The adsorption capacity at equilibrium time, *Q_e_* can be expressed as:

(2) $$Q_{e}\;=\;\frac{\left(C_{0}\;-\;C_{e}\right)}{m}\times V$$

where *m* is the weight of the used dye adsorbent in milligrams (mg), and *V* is the volume of the used dye solution in liters (L) [30,31]. Also, $C_{e}$ represents the dye concentration of the solution at equilibrium. Each test was repeated five times and the values of average and standard deviation (as error bars in the results) are reported. The tests were conducted with 50 mg/L of initial dye concentration and at a temperature of 27 °C. The dye concentration was determined by an ultraviolet-visible (UV–Vis) spectrophotometer (Lambda 25, Perkin Elmer, Boston, MA, USA).

#### 2.3.4. Dye Desorption

Following the completion of adsorption tests, dye desorption of the saturated nanocomposites was performed by immersing them in 50/50 vol % water/ethanol solution at a temperature of 27 °C under shaking for 24 h. Then, the concentration of dye in the solution was measured by an UV–Vis spectrometer to make sure the dye was desorbed. Additional time was given for desorption if it was needed. Adsorbents were rinsed with HCl (hydrochloric acid) and dried before they were used again in the adsorption test. Desorption percentage was calculated as the percent of the desorbed mass with respect to the adsorbed mass. In other words, desorption percent was calculated with considering the weight of the adsorbed dye only, not the total weight of the dye that was initially added in the solution.

#### 2.3.5. Swelling Measurements

Water uptake or swelling amount of the dye adsorbent hydrogels is one of the important parameters in their characterizations. Equation (3) is usually used to calculate the degree of swelling (*DS*) of an absorbent [32]:

(3) $$DS=\frac{W_{s}\;-\;W_{d}}{W_{d}}$$

where *W_d_* and *W_s_* are the weight of the dry and wet states of the dye absorbent, respectively [33]. The swelling behavior of the nanocomposites was examined at different pH levels of water. The pH of the water was controlled by the amount of added NaOH (sodium hydroxide) or HCl (hydrochloric acid) to the solution.

Degree of swelling was determined by immersing 0.1 g of dry adsorbent in 25 mL water. The samples were then brought out from the water and cleaned up with tissue paper to remove surface water. Swollen samples were then weighed and the degree of swelling was calculated using Equation (3).

## 3. Results and Discussion

### 3.1. FTIR Spectroscopy

**Figure 2:**
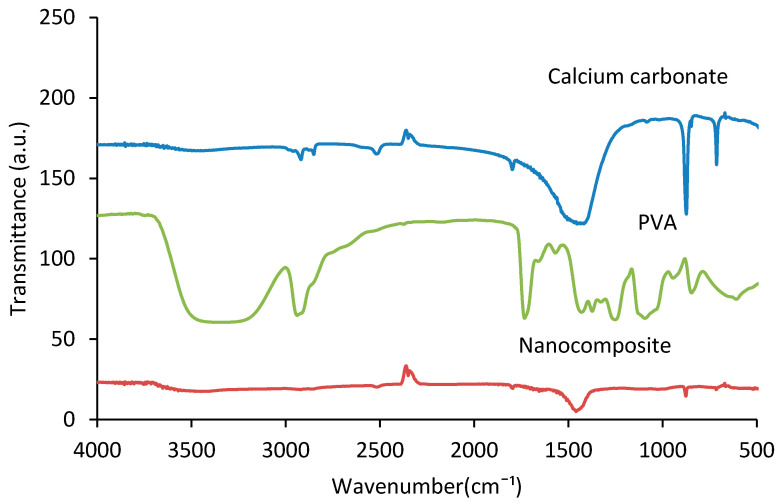
Fourier transform infrared (FTIR) spectroscopy of PVA, calcium carbonate, and PVA/ CaCO_3_ nanocomposites.

Figure 2 shows the FTIR spectroscopy of PVA, calcium carbonate, and the fabricated PVA/CaCO_3_ nanocomposite. The broad peak between 3100 and 3600 cm^−1^ is created by absorbed water molecules [34] or hydroxy groups from PVA, associated with O–H stretch from the intermolecular and intramolecular hydrogen bonds [35]. The absorption peaks at 2910 and 2845 cm^−1^ are corresponding to the stretching of the asymmetric and the symmetric vibration of the CH bond of PVA main chain, respectively. The peaks between 1700 and 1750 cm^−1^ are associated with C–O stretches in PVA. Moreover, the other peaks between 600 and 1500 cm^−1^ are typical characteristic peaks of PVA, associated with (CH)–CH_2_, (OH)–C–OH, (C–O)–C–OH and some other bands in the material [36,37]. In the FTIR spectrum for CaCO_3_, the peaks at 690, 725, 863, and 1065 cm^−1^ are due to crystalline portions of calcium carbonate [38]. The peaks at 709, 855, 1082, 1455, 1786 cm^−1^ are prominent features of the carbonate ions presented in the calcium carbonate. The peaks observed at 855 and 1082 cm^−1^ are symmetrical stretching and wagging of CO_3_^2−^ [39], respectively. By comparing the nanocomposite spectra with the PVA and calcium carbonate spectra, overall the peaks of both PVA and carbonate calcium are present in the nanocomposite, but at different intensity levels. The intensity of carbon peaks, from 690 to 1786 cm^−1^ and 3100 to 3400 cm^−1^ [40], was lower in the nanocomposites, indicating a low amount of PVA in the samples. It should be noted that no new B–O–C peak was detected in the spectra. It was thus hard to say if there was any reaction between PVA and Borax. Also, no reaction between CaCO_3_ and PVA or Borax was apparent. However, CaCO_3_ might have weak electrostatic interaction with the –OH group from PVA; Ca from CaCO_3_ shows as electronic positive and O from –OH shows as electronic negative due to atom polarity. This interaction is however too weak to be detected by FTIR.

### 3.2. Microstructure

**Figure 3:**
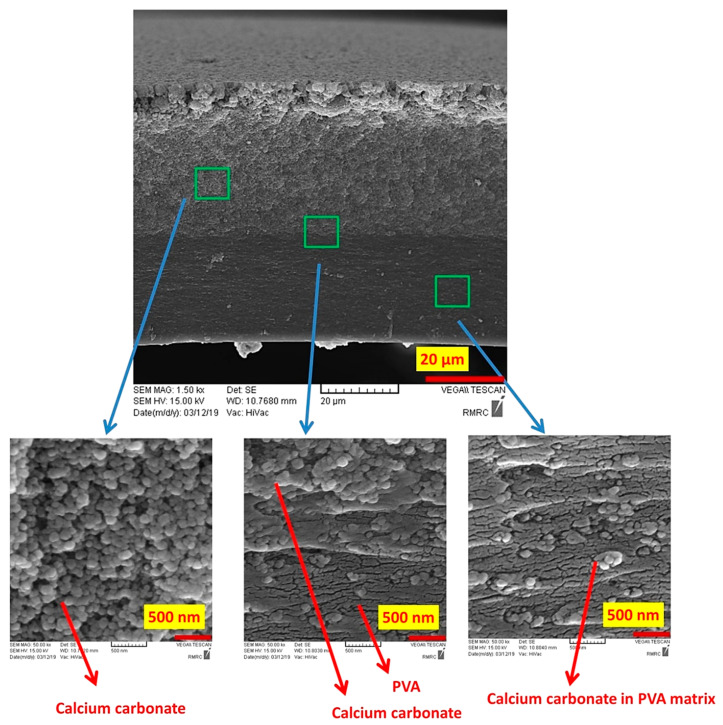
Scanning electron microscopy (SEM) images of PVA/CaCO_3_ nanocomposites showing calcium carbonate rich and PVA-rich zones in the bilayer sample.

Figure 3 shows the SEM images of the fabricated PVA/CaCO_3_ nanocomposite. A bilayer structure of the nanocomposite is noticeable. While CaCO_3_ nanoparticles were densely packed in the top layer, the nanoparticles were dispersed in the PVA matrix at the bottom layer. The formation of a bilayer structure is primarily due to the precipitation of dense CaCO_3_ nanoparticles during drying, their large contact surface area and their high correlation energy. The average diameter of about 50 nm for calcium carbonate nanoparticles and the uniform distribution of the nanoparticles in the matrix of PVA in the bottom layer are visible in Figure 3. This bilayer structure is unique in that the lower layer with high PVA content provides the structural and mechanical integrity to the sample, while the upper layer with a high concentration of calcium carbonate provides an efficient interaction with dyes during the removal process. The crosslinked PVA at the top layer acts as a binder and holds the nanoparticles intact within the overall structure.

### 3.3. Dye Removal and Efficiency

**Figure 4:**
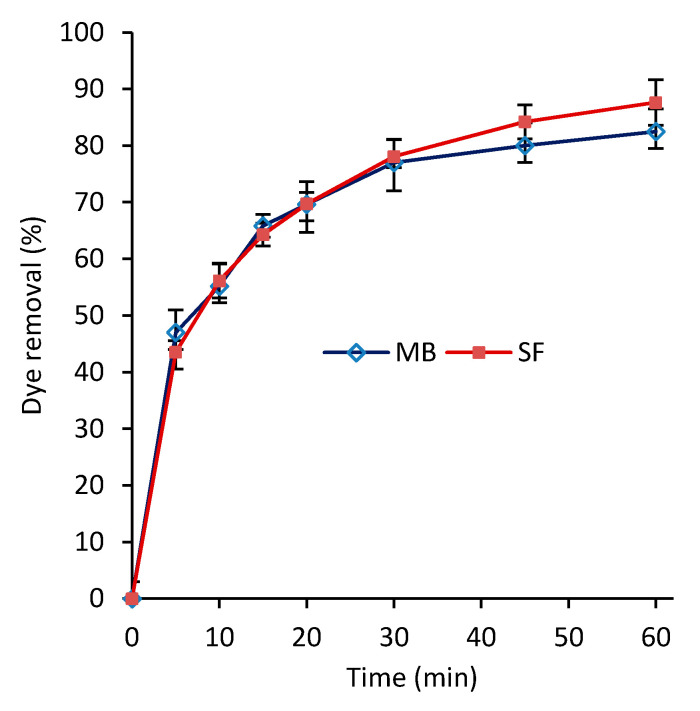
Dye removal percentage versus time for methylene blue (MB) and safranin (SF) (*T* = 27 °C and *C*_0_ = 50 mg/L).

Figure 4 shows the dye removal versus contact time for MB and SF dyes at 27 °C with an initial dye concentration of 50 mg/L. As the time increases, the dye removal percentage increases for both MB and SF dyes with similar rates. As expected, at the early stages of the adsorption (~5 min), the dye removal percentage rapidly increased to ~45%, beyond which the rate slowed down, reaching to 87% and 82% for SF and MB, respectively, after ~60 min. The equilibrium adsorption was reached in about 60 min. Providing an additional 60 min of the adsorption time led to an increase of less than 1% in dye removal percentage for both SF and MB, and hence they were not reported in Figure 4. Our literature review shows that the developed nanocomposite has relatively short equilibrium adsorption time. For instance, Latif et al. [41] reported 200 min for their developed PVA-kaolin-based hydrogel to reach to 80% dye removal. It reached the adsorption equilibrium state after about 350 min with 90% dye removal. In another study, Li et al. [19] reported over 500 min for their developed functionalized PVA with graphene oxide to reach the adsorption equilibrium. A double-layer structure of the developed hydrogel, shown in Figure 3, can be one of the main reasons for the high efficiency of the hydrogel. It is thought that the top layer that contains primarily calcium carbonate nanoparticles acts as an efficient dye adsorbent, and the bottom layer acts as the top layer’s preservative.

### 3.4. Dye Concentration Effect on Adsorption Efficiency

**Figure 5:**
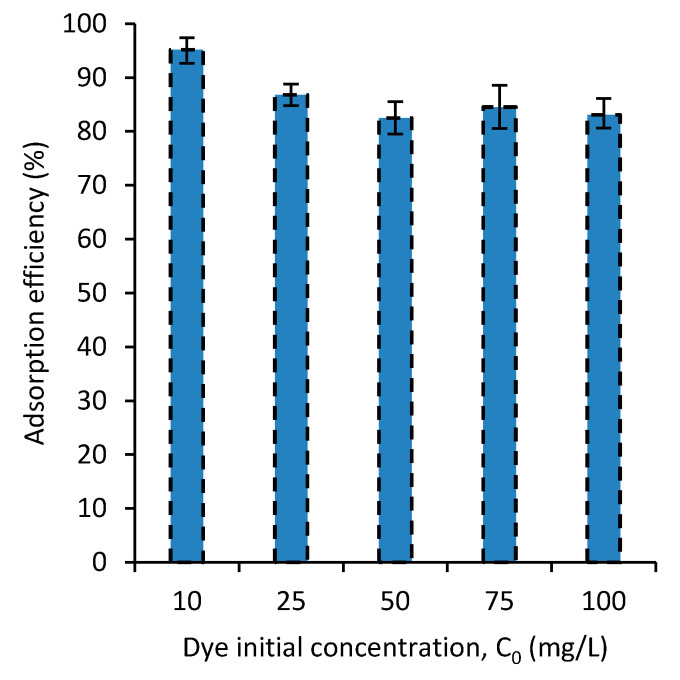
The effect of the initial concentration of MB on the dye removal.

To further understand the adsorption behavior of the dyes by the PVA/CaCO_3_ nanocomposite developed, the dye-adsorption efficiency was experimentally examined at different initial dye concentrations. Figure 5 shows the effect of the initial concentration of MB on the adsorption. The result shows that with an increase in the initial concentration of the dye, the dye-adsorption efficiency decreases. This means that the dye adsorption is occurring while taking some effect from the dye concentration. This can be explained by the adsorption mechanism in which dye concentration has a weak role on the adsorption. The adsorption mechanism will be discussed in Section 3.7. Small differences were observed in the adsorptions of MB and SF, hence the graph for the SF was not reported. It is believed that interaction of nanocomposites with MB and SF due to their cationic properties are the same.

### 3.5. The Effect of Solution pH on Adsorption Efficiency

**Figure 6:**
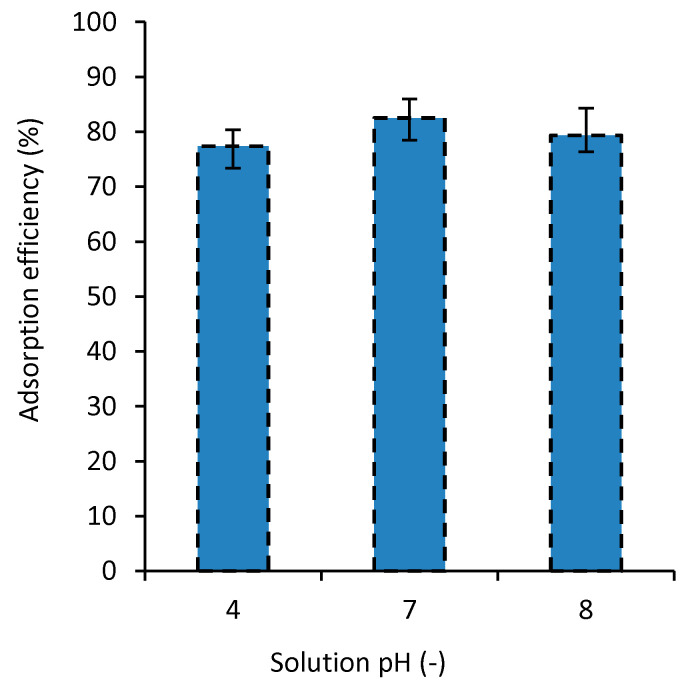
The effect of solution pH on MB adsorption efficiency (*T* = 27 °C and *C*_0_ = 50 mg/L).

Usually, pH plays a significant role on the absorption process [42,43,44,45,46,47]. In general, surface adsorption depends considerably on the pH of the environment [46]. The pH of the solution affects the surface charges of the composite, the degree of ionization of the material, the separation of functional groups from the active positions of the nanocomposite, and the solubility of some pigments. Moreover, how the pH affects the process of dye absorption also depends on both the composite and the dye types [47,48,49]. Hence, the pH of aqueous materials is usually examined when their pollution is investigated. In this study, the effect of the solution pH on the MB dye absorption was investigated on a range for pH, 4 to 8, to cover both acidity and basicity effects. Figure 6 shows the adsorption efficiency for the nanocomposites in solutions with different pH values tested at 27 °C and dye concentration of 50 mg/L. The results of this experiment show that for the pH of 7, the dye adsorption is the highest. When pH becomes 4 or 8, the dye removal decreases about 4.8% and 3.6%, respectively. Ghourbanpour et al. [18] suggested a significantly larger effect of the pH on the efficiency of the dye removal for their developed PVA/Fe(III) and PVA/Chitin/Fe(III) adsorbents.

### 3.6. Swelling

**Figure 7:**
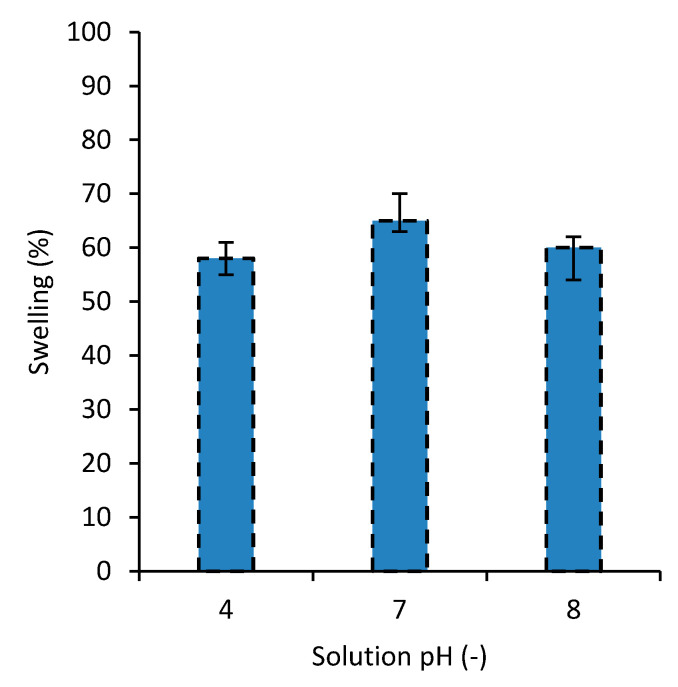
The degree of swelling for the nanocomposite in water with different pH levels.

The swelling behavior of the developed nanocomposites at different pH levels of the water was examined and the results for the degree of swelling are shown in Figure 7. As the results for SF were very close to those of MB, they are not presented here. The results show that the swelling for distilled water (i.e., pH = 7) is the highest. Also the result of this study revealed that, compared with the other PVA-based dye adsorbent hydrogels in the literature [31,34,35], overall, the swelling amount of the developed hydrogel is relatively low [29]. This can be associated with the high surface area of the nanoparticles in the top layer of the nanocomposite. Low swelling is usually considered as an advantage for the dye removal hydrogels, for owning a higher capacity for dye removal. This investigation revealed that the developed hydrogel is able to reach the maximum swelling capacity at a relatively shorter time (i.e., in about 60 min, Figure 4), compared to other PVA-based dye removal hydrogels in the literature [34,35].

### 3.7. Adsorption Mechanisms

**Figure 8:**
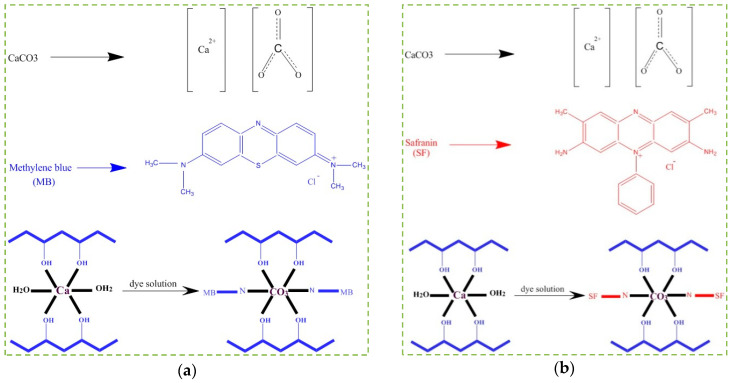
A suggested adsorption mechanism of PVA/CaCO_3_ adsorbent for (**a**) MB, and (**b**) SF.

The absorption is carried out practically in two ways: a) absorption by the presence of soluble particles on the surface of the solid absorbent, and b) absorption by uptake of the particles into the mass of the absorbent [50]. Figure 8 shows the proposed mechanisms for the absorption of MB and SF by PVA/CaCO_3_. This study showed that the most effective dye absorption by PVA/CaCO_3_ was established under neutral conditions (i.e., pH = 7). One hypothesis is that ion-exchange absorption between the centers of Ca^2+^, CO_3_^2−^, MB, and SF governs the absorption mechanism [51]. Ca^2+^ is positively charged, so it is surrounded by OH^−^ of the water. On the other hand, CO_3_^2−^ is attracted by the positive parts of the MB and SF. These attractions result in the separation of MB and SF from water.

### 3.8. Reusability

**Figure 9:**
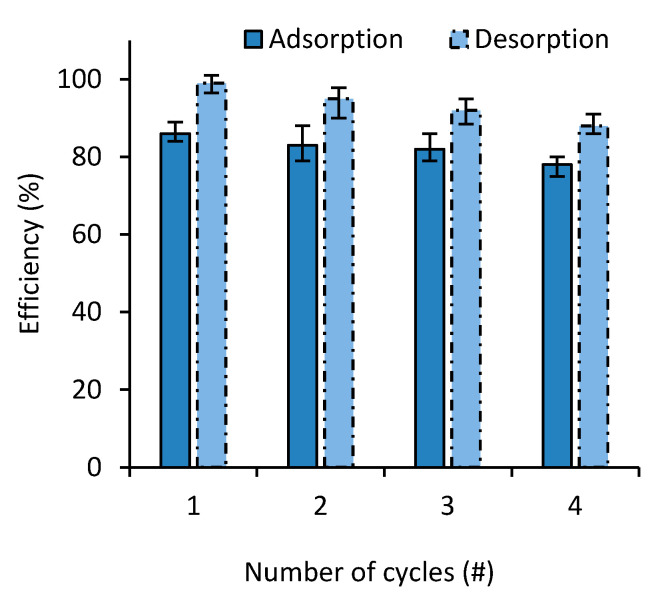
MB adsorption and desorption behaviors on adsorbent over four consecutive cycles.

To investigate the cyclic performance of the prepared nanocomposites, several successive absorption–desorption cycles were performed on both MB and SF. As seen in Figure 9, the adsorbent retained its efficacy with up to four adsorption desorption cycles with MB with less than 9% drop in the adsorption efficiency. FS showed very similar results.

## 4. Modeling

### 4.1. Dye-Adsorption Kinetics

Adsorption kinetics in dye removal is important as it helps in understanding the absorption mechanisms. In recent years, efforts have been made to investigate a series of surface adsorption processes in the removal of pollutants from the environment; efforts have been made to justify their adsorption mechanisms. They are presented in the form of several kinetics models. The proposed models describe the reaction rate of surface adsorption systems based on the concentration of the solution or based on the adsorption capacity. In general, in reaction kinetics, considering the process of changing the chemical properties of a substance over time, the velocity of changes are examined. Since chemical equilibrium is the balance between the reciprocating speeds of a reaction, the fixed concept of equilibrium also derives from kinetic concepts. Therefore, having kinetic data, it is possible to determine equilibrium constants and kinetic constants. Thus, taking into account the dimensions of the reaction site, it is possible to control the time required for the adsorbent to be placed on the adsorbent surface. Hence, knowing this phenomenon is one of the most critical factors in the design of pollutant absorption systems [52,53].

Three kinetic models were considered: a pseudo first-order model, pseudo second-order model, and intraparticle diffusion model. Adsorption capacity as a function of time, *Q_t_* can be stated according to Equations (4)–(6) for nonlinear models of the first-order, the second-order, and the intraparticle diffusion, respectively [54,55,56,57,58,59]. The parameters used in these equations are introduced in Table 1.

(4) $$Q_{t}\left(\frac{\mathrm{mg}}{g}\right)=Q_{e}\;-\;Q_{e}e^{-k_{1}t}$$

(5) $$Q_{t}\left(\frac{\mathrm{mg}}{g}\right)=\frac{k_{2}Q_{e}^{2}t}{1+Q_{e}k_{2}t}$$

(6) $$Q_{t}\left(\frac{\mathrm{mg}}{g}\right)=k_{di}t^{0.5}+D_{i}$$

**Figure 10:**
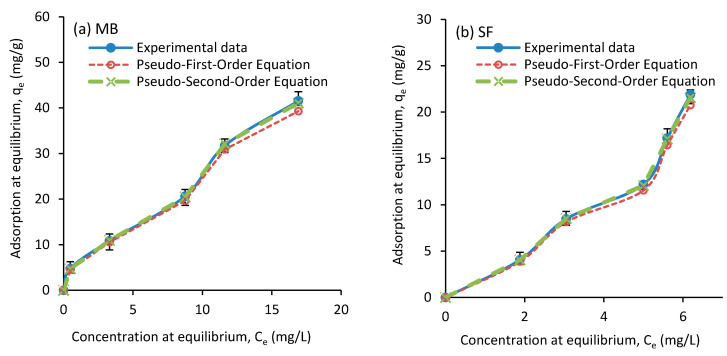
Experimental and pseudo first-order and pseudo second-order kinetic models data of equilibrium absorption for (**a**) MB and (**b**) SF as a function of adsorbent dose.

In this work, the kinetics of the MB dye adsorption at the concentrations of 10, 25, 50, 75, and 100 mg/L and the SF dye at concentrations of 10, 20, 30, 40, and 50 mg/L, both at 27 °C, were investigated. The results of this investigation are presented in Table 2 and Table 3. The results show that the correlation coefficient (R^2^) for both dyes is higher in both first-order and second-order kinetic models (in the range of 0.97 to 0.99), compared to those of intra-particle diffusion model (in the range of 0.64 to 0.91). Tharaneedhar et al. [60] reported a correlation coefficient in a range from 0.95 to 0.97 for those two models for the same dyes. Also, as Figure 10 shows, the values calculated by the pseudo second-order equation do not differ much from the experimental data. Hence, cationic dye MB adsorption on the developed calcium carbonate nanocomposite can be described well using the pseudo second-order model [56]. A similar conclusion can be reached when SF dye was used [61]. In this study, for the intra-particle diffusion model, the correlation coefficient was in a range from 0.64 to 0.91, where with an increase on the concentration, the coefficient was decreased. Tharaneedhar et al. [60] reported a correlation coefficient in a range from 0.963 to 0.969 for this model. It seems that intra-particle diffusion model does not fit well with our experimental results for both MB and SF dyes. This can be due to the structure of the nanocomposite produced.

The dye absorption mechanism consists of the following sequential steps: transfer of the dye molecules from the boundary films to the adsorbent outer surface, intraparticle diffusion of the dye molecules, and the adsorption of dye molecules by the active adsorbents. The dye absorption speed is controlled by the slowest stage, which can be intraparticle penetration or film diffusion [57]. When designing a wastewater treatment process, it is essential to measure the speed of the absorption. According to Weber and Morris, if intraparticle penetration is the speed control factor, the adsorption will be changed with the square root of the time [62]. Therefore, it is possible to measure the rate of the dye adsorption by measuring the adsorption capacity of the adsorbent as a function of the square root of the time.

Kinetic results were also analyzed using intraparticle diffusion to describe adsorption behavior. This model is important because it determines the speed limiting stage in liquid phases. The parameters of the models were calculated and presented in Table 2 and Table 3. The results for the intraparticle diffusion model show that the penetration rate of MB and SF dye into the adsorbent nanoparticles increased with increasing dye concentration due to a constant increase in the intraparticle diffusion coefficient, *k_di_*. The results of this study also show that PVA/CaCO_3_ nanocomposite is a suitable adsorbent for absorbing MB and SF dyes in low and medium concentrations.

### 4.2. Isotherm Adsorption

To investigate the type of interaction between gravity and the dye, the adsorption was evaluated using two known models, the Langmuir and Freundlich isotherm models. The non-linear expression for the Langmuir and Freundlich isotherm models are presented in Equations (7) and (8), respectively [63,64]:

(7) $$Q_{e}=\frac{Q_{m}K_{L}C_{e}}{1+K_{L}C_{e}}$$

(8) $$Q_{e}=k_{f}C_{e}^{1/n_{f}}$$

The parameters in Equations (7) and (8) are introduced in Table 4.

In the Freundlich model, if *n* = 1, the space between the two phases is dye concentration-independent. For a usual absorbance, 1/n is less than 1. The equilibrium parameter (*R*L), the Langmuir equilibrium main characteristic, can be expressed using Equation (9), where the *R*L value indicates the desired Langmuir equilibrium (0 < *R*L < 1), linear (*R*L = 1), undesirable (*R*L > 1) or irreversible (R = 0) [63].

(9) $$RL=\frac{1}{1+K_{L}C_{h}}$$

**Figure 11:**
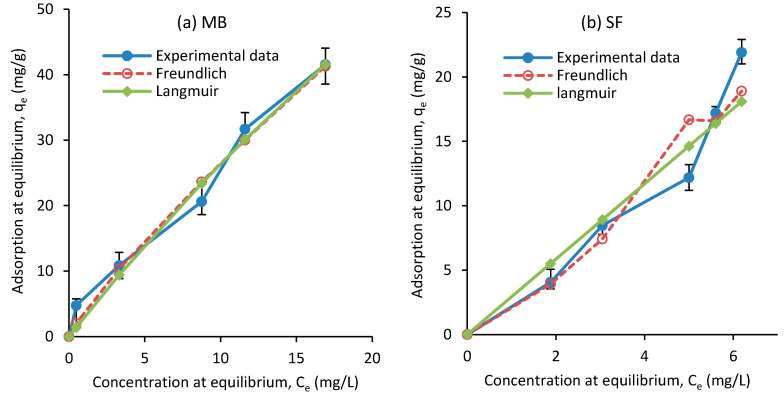
Isotherm adsorption of (**a**) MB and (**b**) SF on the adsorbent obtained experimentally and from the Langmuir and Freundlich models.

In Equation (9), $C_{h}$ indicates the highest color concentrations. Figure 11 shows the adsorption values from the experimental data, and Langmuir and Freundlich models. The constant parameters were calculated based on the experimental data for the isotherm models and are listed in Table 5. The results show that the correlation coefficient of the isothermal equation for the Freundlich model is relatively high. Therefore, it can be said that the removal of MB and SF dyes in several layers follows the isothermal equation of Freundlich [64]. The Freundlich adsorption isotherm model was introduced for single-layer absorption and heterogeneous absorption sites with unequal energies. When $k_{f}$ increases, the equilibrium adsorption of the adsorbent increases. According to the constant values of *R*L for adsorption (Table 5), the Langmuir model is also suitable and desirable for this adsorption. The values reported for *Q_m_* in the literature are in a wide range. While Othman et al. [65] suggested 1666.67 mg/g for the removal of MB dye, Tharaneedhar et al. [60] reported 82.71 mg/g and Gourbanpour et al. [18] obtained 810.4 mg/g for their developed composite. For SF dye removal, Abukhadra et al. [66] and Sahu et al. [67] reported 65.35 and 89.47 mg/g, respectively. The *Q_m_* values obtained in this work are 249.9 and 81.83 mg/g for MB and SF, respectively, both of which are within the range of reported values.

## 5. Conclusions

A polyvinyl alcohol/calcium carbonate nanocomposite was developed using a facile water-based solution method and was used as an adsorbent for dye removal. Two cationic dyes, i.e., methylene blue and safranin cationic dyes, were used for the adsorption and desorption tests. SEM micrographs showed a bilayer structure; one rich in calcium carbonate and the other rich in polyvinyl alcohol. Kinetic studies showed that the dye-adsorption process is well matched to the pseudo second-order kinetic model. An isotherm adsorption study showed that experimental data have the best compliance with the Freundlich isotherm model. The results revealed that the developed nanocomposite has relatively high adsorption efficiency and short adsorption time and retains its performance after several successive absorption–desorption processes. Based on the results of this study, calcium carbonate can potentially be used as an efficient nanomaterial filler in a PVA matrix for the removal of cationic dyes from aqueous solutions. This high capability of PVA/CaCO_3_ nanocomposites in decontaminating wastewater containing cationic dyes provides a potential for it to be used in wastewater treatment in the textile industry without the need for high pressure or high heat which is commonly used.

## Figures and Tables

**Table 1:** Parameters used in Equations (4) to (6).

Used Parameters in Equation (4)	Used Parameters in Equation (5)	Used Parameters in Equation (6)
t (min): time	t (min): time	t (min): time
$k_{1}\;\left(\frac{1}{\min}\right)$: Pseudo-first-order kinetic coefficient	$k_{2}\;\left(\frac{g}{\mathrm{mg}.\min}\right)$: Pseudo-second-order kinetic coefficient	$k_{di}\;\left(\frac{\mathrm{mg}}{g.\min^{0.5}}\right)$: intraparticle diffusion coefficient
$Q_{e}$ (mg/g): adsorption efficiency or equilibrium adsorption	$Q_{e}$ (mg/g): adsorption efficiency or equilibrium adsorption	$D_{i}\;\left(\mathrm{mg}/g\right)$: intercept for boundary layer thickness

**Table 2:** Experimental data and model parameters for the adsorption of MB from pseudo first-order kinetic, pseudo second-order kinetic, and intraparticle diffusion models at different dye concentrations.

Concentration	Experimental	Pseudo First-Order Kinetic Model			Pseudo Second-Order Kinetic Model			Intra-Particle Diffusion Model		
(mg/L)	$Q_{e}$ (mg/g)	$Q_{e}$ (mg/g)	$k_{1}$ (min^−1^)	R^2^	$Q_{e}$ (mg/g)	$k_{2}$ (min^−1^)	R^2^	$c_{i}$	$k_{di}$	R^2^
10	4.76 ± 0.12	4.49	0.18	0.98	4.70	34.81	0.99	2.44	0.35	0.91
25	10.85 ± 0.16	10.54	0.11	0.98	10.94	252.80	0.99	2.99	1.23	0.94
50	20.63 ± 0.63	19.65	0.14	0.97	20.46	2132.01	0.99	8.19	1.91	0.94
75	31.70 ± 0.88	30.88	0.13	0.98	32.11	7685.08	0.99	11.60	3.23	0.91
100	41.55 ± 0.95	29.23	0.18	0.97	41.07	23,260.09	0.99	21.12	3.16	0.64

**Table 3:** Experimental data and model parameters for the adsorption of SF from pseudo first-order kinetic, pseudo second-order kinetic, and intraparticle diffusion models at different dye concentrations.

Concentration	Experimental	Pseudo First-Order Kinetic Model			Pseudo Second-Order Kinetic Model			Intraparticle Diffusion Model		
(mg/L)	$Q_{e}$ (mg/g)	$Q_{e}$ (mg/g)	$k_{1}$ (min^−1^)	R^2^	$Q_{e}$ (mg/g)	$k_{2}$ (min^−1^)	R^2^	$c_{i}$	$k_{di}$	R^2^
10	4.06 ± 0.12	3.87	0.12	0.99	4.04	13.36	0.99	2.35	0.03	0.76
20	8.47 ± 0.26	8.14	0.14	0.98	8.50	148.41	0.99	5.39	0.06	0.76
30	12.18 ± 0.32	11.55	0.15	0.98	12.08	459.92	0.99	7.91	0.08	0.77
40	17.19 ± 0.43	16.43	0.13	0.98	17.12	1187.32	0.99	10.75	0.13	0.80
50	21.90 ± 0.65	20.76	0.11	0.97	21.56	2022.21	0.99	12.43	0.18	0.85

**Table 4:** Parameters used in Equation (7) to (8).

Used Parameters in Equation (7)	Used Parameters in Equation (8)
$C_{e}\;\left(\frac{\mathrm{mg}}{L}\right)$: equilibrium concentration	$C_{e}\;\left(\frac{\mathrm{mg}}{L}\right)$: equilibrium concentration
$k_{L}\;\left(\frac{L}{\mathrm{mg}}\right)$: Langmuir constant	$k_{f}\;\left(\frac{L}{\mathrm{mg}}\right)$: Freundlich constant indicating adsorption capacity at unit concentration
$Q_{m}\;$(mg/g): equilibrium adsorption or the maximum absorption capacity	$n_{f}\;$(mg/g): Freundlich constant indicating adsorption intensity

**Table 5:** Constant parameters of the Langmuir and Freundlich models for adsorption of MB and SF.

Dye	Langmuir				Freundlich		
	*k_L_* (L/mg)	*Q_m_* (mg/g)	R^2^	*R*L	*k_f_* ((mg/g)(L/mg))	1/*n_f_*	R^2^
MB	0.0118	249.9	0.97	0.83	3.795	0.85	0.97
SF	0.00035	81.83	0.81	0.99	1.075	1.32	0.84
